# Performance comparison of a new automated cuff pressure controller with currently available devices in both basic research and clinical settings

**DOI:** 10.1186/s40560-016-0126-7

**Published:** 2016-01-12

**Authors:** Junichi Michikoshi, Shigekiyo Matsumoto, Hiroshi Miyawaki, Harushi Niu, Katsuhiro Seo, Makoto Yamamoto, Shu-ichi Tokunaga, Takaaki Kitano

**Affiliations:** Department of Anesthesiology and Intensive Care, Faculty of Medicine, Oita University, 1-1 Idaigaoka-Hasamamachi, Yufu City, Oita 879-5593 Japan; Department of Medical Technologists, Division of Engineering, Kokura Memorial Hospital, 3-2-1 Asano-Kokura Kitaku, Kitakyushu City, Fukuoka 802-8555 Japan; Department of Anesthesiology and Intensive Care Medicine, Kokura Memorial Hospital, 3-2-1 Asano-Kokura Kitaku, Kitakyushu City, Fukuoka 802-8555 Japan; Oita Kyowa Hospital, 953-1 Miyazaki, Oita City, Oita 870-1133 Japan; Tokuki Giken Kogyo Co., Ltd., 318 Onegawa, Usa City, Oita 879-0232 Japan

**Keywords:** Airway management, Mechanical ventilation, Respiratory care, Automated cuff pressure gauge, Tracheal intubation, Cuff pressure

## Abstract

**Background:**

The management of tracheal tube cuff pressure in patients receiving mechanical ventilation is important for the prevention of ventilator-associated pneumonia. Currently, cuff pressure is intermittently monitored with a pressure gauge and adjusted when necessary in a routine practice. However, this method results in wide variations in pressure, and adequate management is difficult due to the spontaneous release of air from the cuff, which reduces cuff pressure. In order to continuously maintain a uniform cuff pressure, we developed a new automated cuff pressure controller and compared its properties with existing devices.

**Methods:**

The effectiveness of the new device was assessed with a model trachea/lung and tracheal tube by measuring cuff pressure while on mechanical ventilation. An electrically powered automatic cuff controller or manual cuff pressure control was used for comparison purposes. The effectiveness of the new device was also examined in patients receiving mechanical ventilation by continuously measuring cuff pressure for a 24-h period.

**Results:**

Cuff pressure was uniformly maintained with the new device. Moreover, in the clinical setting, variation in pressure from the set pressure was minimal with both the new device and existing device, relative to the intermittent monitoring method. This suggests that, as with the existing device, uniform cuff pressure management is possible with the new device.

**Conclusions:**

Our results demonstrate the ability of the new cuff pressure controller to manage cuff pressure without the need of a power source, highlighting its potential utility in clinical settings.

## Background

The management of tracheal tube cuff pressure is an important factor in mechanical ventilation from the perspective of preventing ventilator-associated pneumonia (VAP) [1, 2]. Maintaining appropriate cuff pressure is important, since the cuff plays an important role in compensating for the amount of ventilation and preventing leakage of secretions around the cuff into the respiratory tract. This aspect of airway management is rapidly gaining interest in the clinical setting.

The current method for managing cuff pressure involves intermittently monitoring and adjusting cuff pressure with the aid of a pressure gauge. The goal is to maintain a pressure of 20–30 cmH_2_0 (minimal leak pressure) to minimize damage to the respiratory tract mucosa [3]. However, cuff pressure varies widely when caring for patients, such as during tracheal suctioning, oral care, and patient positioning [4]. Maintaining a certain cuff pressure is particularly difficult in clinical settings due to situations in which spontaneous air leakage from the cuff reduces pressure [5].

To address the above issues, an automated cuff pressure controller was developed recently (2014) and is currently being used in the clinical setting [6]. Many existing devices use a system of electronically monitoring cuff pressure and deflating/inflating as necessary. While a particular pressure can be maintained over a long period with these devices, they lack the ability to adjust to sudden changes in pressure, and their reliance on electricity also detracts from convenience. Given this, we developed and confirmed the safety of a new automated cuff pressure controller (Cuff-Keeper), which was based on the concept of minimizing pressure variations from the set pressure and allowing for continuous management in the absence of a power source.

The new device was compared to a traditional automated cuff pressure controller and the routine intermittent monitoring method, as well as existing commercially available devices. First, we compared the effectiveness of the new automated cuff pressure controller with the currently available device using 24-h cuff pressure variation and the pressure load test. In the next study, we examined the utility of the new automated cuff pressure controller by continuously recording changes in cuff pressure in patients receiving mechanical ventilation by each device.

## Methods

### New automated cuff pressure controller

We describe here a new automated cuff pressure controller, which was developed based on the concept of a durable device that does not require a power source, and can continuously maintain uniform cuff pressure, while also being able to rapidly adjust to sudden pressure changes. The device was developed in collaboration with Tokuki Giken Kogyo Co., Ltd. (Oita, Japan).

**Fig. 1:**
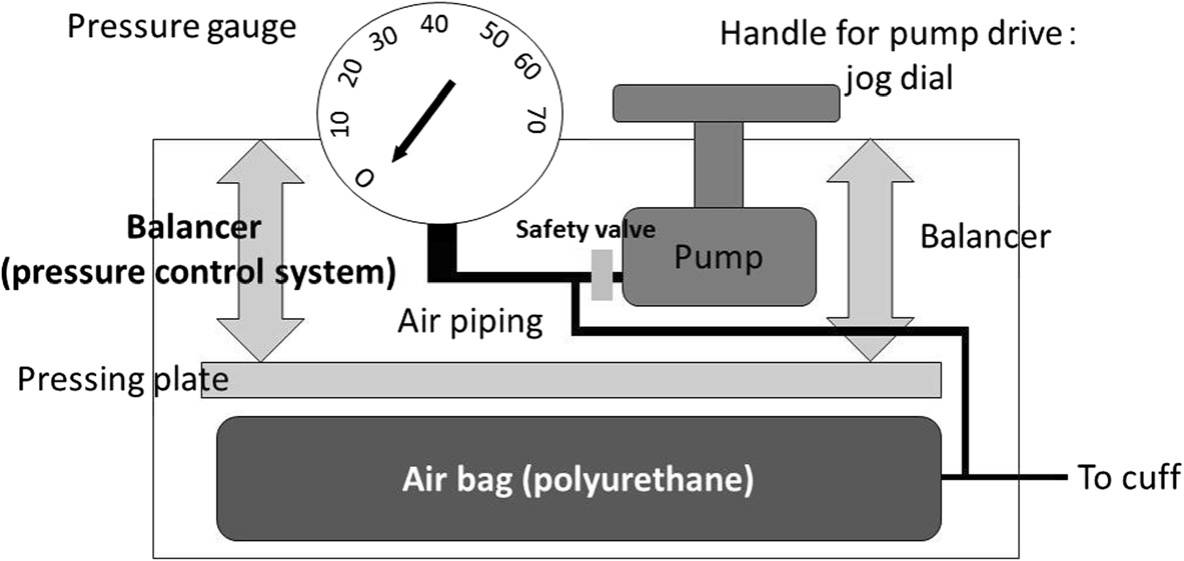
Device schematic. Structural diagram of the new automated cuff pressure controller

In order to deal with sudden pressure changes, the new device uses a pressure control system with an air bag and pressing plate. The device comprises an air bag, pressing plate, pressure control system, air pump, jog dial, safety valve, and pressure gauge (Fig. 1). When cuff pressure suddenly changes due to the angle of the neck or bucking during mechanical ventilation, the pressing plate immediately works to stabilize the pressure, and through the flow of air from the cuff into the air bag, pressure on the respiratory tract mucosa is absorbed. For example, if the size of the air bag is 100 mm × 50 mm × 10 mm, and 1 ml (1000 mm^3^) of air escapes from the cuff, the change in air bag height (H) becomes H =1000/(100 × 50) = 0.2 mm. Through the pressure control system, the pressing plate is displaced toward the cuff side by 0.2 mm, allowing for proper adjustment of pressure. On the other hand, if pressure is applied to the cuff and 1 ml of air enters to the air bag, the pressing plate is displaced upward by 0.2 mm. Changes in cuff pressure can be minimized through this principle.

The new device is easy to use—by turning the jog dial, the cuff is inflated through the inflow of air from within the circuit. Pressure is maintained once the cuff pressure reaches the set value and air inflow from the pump is stopped. The pump has a safety valve that protects the pressure gauge from damage resulting from air inflow when the jog dial is turned excessively. The device has an open atmospheric valve, with an upper pressure limit of 70 cmH_2_O and a lower limit of −10 cmH_2_O.

The system described above allows for the continuous management of cuff pressure without a power source. The device has been approved by the Ministry of Health, Labour, and Welfare of Japan, and is currently available commercially.

### Equipment

The following equipment were used in this study: pneumatic-driven automatic cuff pressure controller (Cuff-Keeper; Tokuki Giken Kogyo Co., Ltd.; hereafter, “device A”), which is included in the new cuff pressure controller; electrically powered automatic cuff controller (COVIDIEN, Ireland; hereafter, “device B”); and manual cuff pressure control (COVIDIEN, Ireland; hereafter, “device C”), which is used in conjunction with the intermittent monitoring method.

### 24-h cuff pressure variation

A model polyvinyl chloride-based trachea with an 18 mm inner diameter was connected to a model lung (Training/Test Lung “TTL”; Michigan Instruments, USA), and a tracheal tube (Taper Guard Evac Oral Tracheal Tube; COVIDIEN, Ireland) with an inner diameter of 8.5 mm was inserted into the trachea. The PB840 mechanical ventilator (COVIDIEN, Ireland) was set on the A/C mode of volume-control ventilation (VCV) with the following parameters: tidal volume, 500 ml; breathing, 15 times/min; PEEP, 3 cmH_2_O. The cuff pressure of the devices was set to 20 cmH_2_O. Temporal changes in inner cuff pressure were measured over the course of 24 h. To measure inner cuff pressure, the pilot balloon of the tracheal tube was connected to a 3-way stopcock, one side of which was connected to one of the devices and the other to a blood pressure transducer (TruWave Transducer; Edwards Lifesciences, CA, USA), which was in turn connected to a bedside monitor (BSM-2301; Nihon Kohden, Tokyo, Japan). Data were collected through a central monitor (CNS-9601; Nihon Kohden, Tokyo, Japan).

Coefficient of variation (CV) values were used to compare pressure changes via recorded mean values of maximum peak pressure and minimum peak pressure during a 1-min period. Each experiment was performed a total of ten times.

### Pressure load test

In order to evaluate how each device responded to sudden changes in pressure, 5 and 20 g weights were dropped from a height of 10 cm onto the top of the cuff and the resulting changes in cuff pressure were recorded and analyzed. A flow analyzer (PF-300; IMI, Aichi, Japan) was connected to the pilot balloon, and changes in pressure were recorded, as was the time it took for pressure to return to the set pressure. Results from this examination were compared between device A, device B, and device C. Each experiment was performed a total of ten times.

### Comparison of maximum cuff pressure and the number of times cuff pressure exceeded 35 cmH_2_O

The ethics committee of Kokura Memorial Hospital (Kitakyushu, Fukuoka, Japan) approved this study. Informed written consent was obtained from all patients recruited to the study. Continuous cuff pressure management was performed in patients receiving mechanical ventilation, and cuff pressure was recorded over time and changes were compared between devices. New device A was compared with existing device B and routine intermittent monitoring with device C.

The present study targeted cases that received mechanical ventilation maintenance between March and May of 2014 at the CCU of Kokura Memorial Hospital. Each case was randomly assigned to a treatment group prior to data collection. Those who had undergone tube removal within 24 h of tracheal intubation and those who died were excluded from the study. Measurements were taken in patients receiving mechanical ventilation. Since a number of patients required extubation during measurements, measurements with the three devices could not be obtained for some patients. The final number of patients assessed for each device was as follows: device A (10 patients), device B (10 patients), and device C (7 patients).

Cuff pressure was set at 18–25 cmH_2_O, and corrective adjustments were made once per day with device A, 0 times/day with device B, and 12 times/day with device C. The pilot balloon and cuff pressure gauge were connected by tubing via a 3-way stopcock, connected to the patient monitor (Nihon Kohden, Japan) cable through a blood pressure transducer (TruWave transducer; Edwards Lifesciences, CA, USA). Using the method described above, we measured the cuff pressure of each device continuously for 24 h. Cuff pressure varied when patients coughed or changed their position. In order to determine whether there were differences between the devices in terms of the ability to maintain cuff pressure, we determined the maximum cuff pressure of each device during measurements. Moreover, given that cuff pressure was observed to suddenly change within a few seconds, we counted the total number of times the cuff pressure suddenly exceeded >35 cmH_2_0 and returned to 18–25 cmH_2_O over a 24-h period.

### Statistical analysis

Data were analyzed by Kruskal-Wallis one-factor analysis of variance (ANOVA) and the multiple comparison test (Bonferroni/Dunn) using StatView-J5.0. Data are presented as mean ± SD. *P* < 0.05 was considered significant.

## Results

### 24-h average cuff pressure

**Fig. 2:**
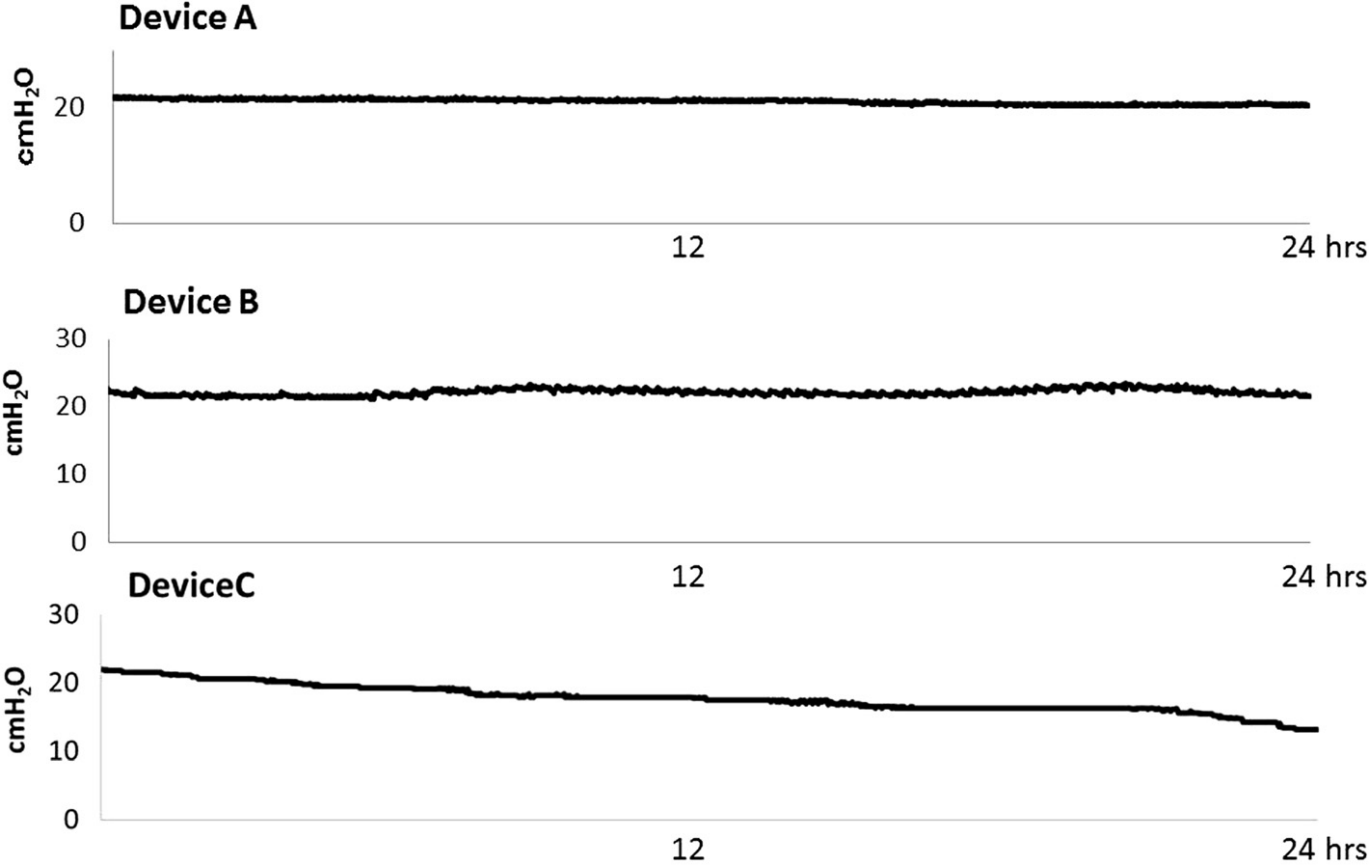
Representative traces from continuous measurements of cuff pressure over a 24-h period using a model trachea/lung system. Representative traces are shown for the newly developed automated cuff pressure controller (Cuff-Keeper, Tokuki Giken Kogyo, Oita, Japan; device A), the automated cuff pressure controller (COVIDIEN, Ireland; device B), and the intermittent monitoring method (COVIDIEN, Ireland; device C). Values are the means of every minute. Device A, 21.0 ± 0.4 cmH_2_O (CV 2.0 %); device B, 22.1 ± 0.4 cmH_2_O (CV 2.1 %), and device C, 17.4 ± 1.7 cmH_2_O (CV 10.0 %). *CV* coefficient of variation

A tracheal tube was inserted into a model trachea/lung and connected to a mechanical ventilator, followed by initiation of ventilation. Changes in cuff pressure over a 24-h period were compared between devices. The average cuff pressure for each of the three devices were as follows: device A, 21.0 ± 0.4 cmH_2_0; device B, 22.1 ± 0.4 cmH_2_0; device C, 17.4 ± 1.7 cmH_2_0. When comparing average cuff pressure relative to the set cuff pressure by CV values, device A had the least variation (device A, 2.0 %; device B, 2.1 %; device C, 10.0 %; Fig. 2).

### Pressure load test

**Fig. 3:**
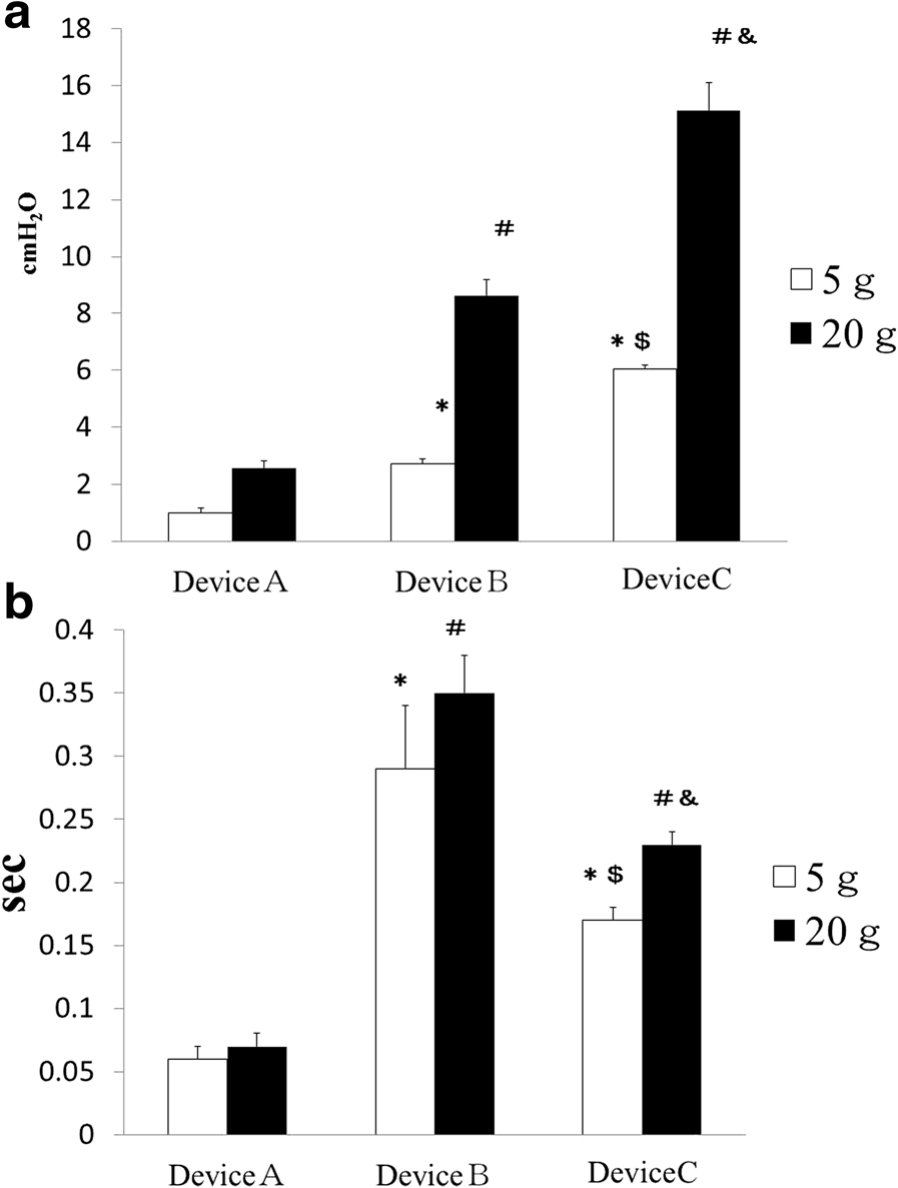
Performance comparison of the new automated cuff pressure controller with currently available devices. **a** Mean cuff pressure variation ± SD in response to the impact from weight dropping is shown for the newly developed automated cuff pressure controller (Cuff-Keeper, Tokuki Giken Kogyo, Oita, Japan; device A), automated cuff pressure controller (COVIDIEN, Ireland; device B), and intermittent monitoring method (COVIDIEN, Ireland; device C). *White bars* indicate that 5 g weights were dropped from a height of 10 cm onto the top of the cuff. *Black bars* indicate that 20 g weights were dropped from a height of 10 cm onto the top of the cuff. *p < 0.0001 vs. device A 5 g. ^#^ *p* < 0.0001 vs. device A 20 g. ^$^ *p* < 0.001 vs. device B 20 g. **b** Mean time ± SD for cuff pressure to return to the set pressure after weight dropping is shown for the newly developed automated cuff pressure controller (Cuff-Keeper, Tokuki Giken Kogyo, Oita, Japan; device A), automated cuff pressure controller (COVIDIEN, Ireland; device B), and intermittent monitoring method (COVIDIEN, Ireland; device C). *White bars* indicate that 5 g weights were dropped from a height of 10 cm onto the top of the cuff. *Black bars* indicate that 20 g weights were dropped from a height of 10 cm onto the top of the cuff. *p < 0.0001 vs. device A 5 g. ^#^ *p* < 0.0001 vs. device A 20 g

The results of the weight dropping test are shown in Fig. 3a, b. The average cuff pressures were significantly smaller with both the 5 and 20 g weights for device A compared to devices B and C. The range of variation in response to sudden changes in pressure was also small for device A.

The time required to return to the set pressure was significantly shorter for device A compared to devices B and C with both the 5 and 20 g weights. These results suggest that the new device more efficiently adjusts to pressure changes compared to the existing devices.

### Clinical use

Table 1 presents demographic data. The utility of devices A and B, as well as intermittent monitoring with device C, was evaluated in patients receiving mechanical ventilation.

**Table 1:** Demographic data

	Device A	Device B	Device C
	*N* = 10	*N* = 10	*N* = 7
Sex, male (%)	10 (100)	10 (100)	7 (100)
Age (mean ± SD)	72.1 ± 8.4	72.3 ± 8.1	72.1 ± 9.5
Diagnostic group (%)			
Cardiology			
Congestive heart failure	2 (20)	2 (20)	1 (14)
Aortic dissection	1 (10)	1 (10)	0 (0)
Myocardial infarction	2 (20)	1 (10)	2 (29)
Cardiopulmonary arrest	5 (50)	6 (60)	4 (57)
APACHE-II score			
(mean ± SD)	20.6 ± 4.6	21.3 ± 4.1	20.7 ± 4.8
SOFA score			
(mean ± SD)	7.6 ± 2.2	7.5 ± 2.2	7.7 ± 2.2

#### Comparison of maximum cuff pressure

**Fig. 4:**
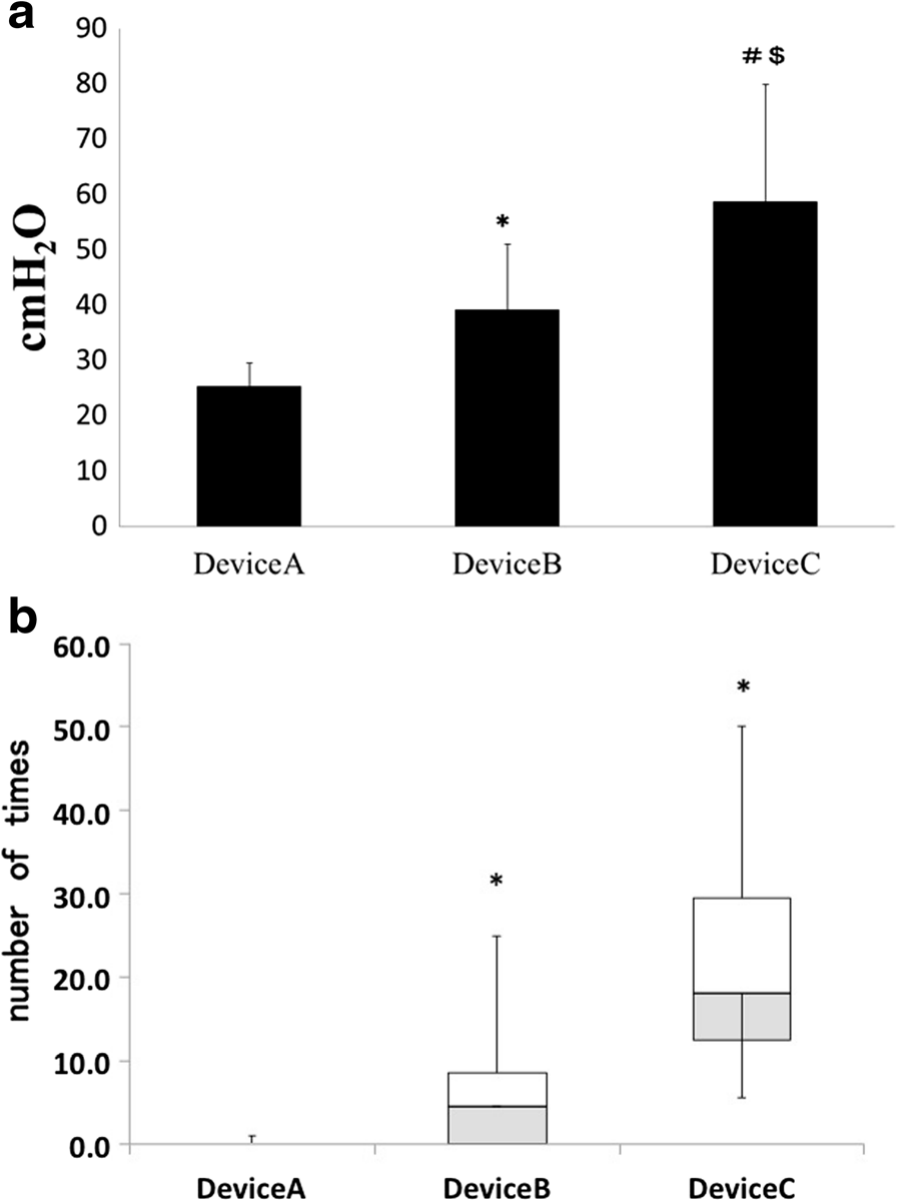
Performance comparison of the new automated cuff pressure controller with the currently available device in a clinical setting. **a** The peak cuff pressure value for the newly developed automated cuff pressure controller (Cuff-Keeper), automated cuff pressure controller, and intermittent monitoring method as measured by a cuff pressure gauge. *p < 0.0001 vs. device A max value. ^#^ *p* < 0.0001 vs. device B max value. **b** Number of times the cuff pressure exceeded 35 cmH_2_O over a 24-h period using the newly developed automated cuff pressure controller (Cuff-Keeper), automated cuff pressure controller, and intermittent monitoring method with a cuff pressure gauge. The data are shown as boxes with the central mark indicating the median. The edges of the box are the 25th and 75th percentiles. The whiskers show the minimum and maximum values. *p < 0.0001 vs. device A. ^#^ *p* < 0.0001 vs. device B

The mean maximum cuff pressure ± SD obtained from trend graphs for devices A, B, and C were 25.3 ± 4.2, 39.2 ± 11.8, and 58.7 ± 21.1 cmH_2_O, respectively (Fig. 4a). These results suggest that device A better maintained uniform cuff pressure compared to device B in response to sudden increases in cuff pressure, and that the intermittent monitoring method showed substantial variation in pressure.

#### Comparison of the number of times when cuff pressure exceeded 35 cmH_2_O

The number of times when cuff pressure exceeded 35 cmH_2_O was obtained from trend graphs for devices A, B, and C (Fig. 4b). Compared to devices B and C, device A had a significantly lower number of times when cuff pressure exceeded 35 cmH_2_O. Similar to the results for maximum cuff pressure, devices A and B were able to maintain uniform cuff pressure in response to sudden decreases in cuff pressure, while the intermittent monitoring method showed substantial variation.

## Discussion

We assessed the safety of a new automated cuff pressure controller, which was developed for use in clinical settings based on the concept of continuous cuff pressure management in the absence of a power source, while maintaining cuff pressure near the set pressure in response to various perturbations. The utility of the device for continuous cuff pressure management was demonstrated by recording changes in cuff pressure over time in patients receiving mechanical ventilation.

Maintaining adequate cuff pressure during mechanical ventilation is important for preventing leakage of secretions around the cuff into the respiratory tract, as well as compensating for the amount of ventilation and preventing VAP [1]. The intermittent monitoring method involves monitoring pressure with a pressure gauge and maintaining it at 20–30 cmH_2_0 (minimal leak technique) in order to minimize damage to the respiratory tract mucosa [3], but tracheal suctioning, oral care, and patient positioning can cause marked changes in cuff pressure. The situations that result in the spontaneous release of air from the cuff pose challenges in clinical settings. We demonstrated the limitations of the intermittent monitoring method, with which pressure variation can be substantial. We believe that it will be important for the cuff pressure adjusting devices to play a part in cuff pressure management in the future [7–10].

Our new device demonstrated better maintenance of uniform cuff pressure than the existing devices when continuously measuring cuff pressure over a 24-h period using a model lung/trachea system. In the weight-drop test as well, the new device showed significantly less pressure variation (CV) compared to the existing devices. The new device utilizes a system in which the cuff of the tracheal tube and air bag, and pressure control system involving a pressing plate, work together to adjust to sudden changes in pressure. This, in turn, allows the device to absorb pressure applied to the cuff. In addition, the new device does not have a pump function to adjust for decreases in cuff pressure, and a pressure control system based on an air bag has its limitations. However, by demonstrating that the cuff pressure was below the set pressure for a short time, and by periodically monitoring the pressure, the new device can be used without issues in the clinical setting. Our results demonstrate that the new device provides superior uniform cuff management over existing devices.

We also found that changes in maximum cuff pressure, as well as the times when cuff pressure exceeded 35 cmH_2_O, were significantly lower with the new device compared to existing devices. Similar results were obtained when comparing results from the weight-drop test. The maximum cuff pressure and the number of times when cuff pressure exceeded 35 cmH_2_O reflect the response to sudden rises in pressure. During mechanical ventilation, various events can trigger sudden changes in pressure, such as tracheal suctioning, patient positioning, oral care, and bucking. Consistent with this, severe pressure fluctuations were observed with the intermittent monitoring method. These results collectively demonstrate the utility of the new cuff pressure controller, which efficiently minimized changes in cuff pressure.

An important aspect of cuff pressure management is maintenance of the set pressure. When comparing the proportion of time spent below the set pressure, the automated cuff pressure controller (B) device was superior to the other devices, although the new device was also able to maintain cuff pressure within an acceptable range. This may reflect the fact that this device (B) has an electric pump that allows it to maintain the set pressure, whereas the new device lacks an automatic correction function. While the new device requires periodic monitoring, it has a lock function on the pump that minimizes the release of pressure from the device, allowing for pressure to be maintained within −1 cmH_2_0 of the set pressure over a 24-h period. For this reason, monitoring of pressure once every 24 h for the release of air is sufficient, a task well within the bounds of what is feasible in clinical settings. On the other hand, with the intermittent monitoring method, cuff pressure remained below the set pressure. These results collectively suggest that, when comparing cuff management with the various devices, cuff pressure can be unstable depending on the method/device used.

This highlights the limitations of intermittent monitoring, and underscores the need to use an automated device, when possible.

Our study has several limitations. First, the clinical sample size was small. Moreover, since long-term clinical results are not yet available with the new device, we could not confirm its long-term utility and efficacy. Second, we have not assessed the ability of the automated devices to improve patient clinical status. Further studies will be needed to evaluate these issues and clarify the benefits and drawbacks of using automated devices in clinical settings.

## Conclusions

Our findings highlight the difficulty of adjusting tracheal tube cuff pressure with the intermittent monitoring method currently used in routine practice, as compared with the use of automated devices. Appropriate management of cuff pressure holds the promise of preventing complications such as aspiration that result from secreted material entering the respiratory track when cuff pressure decreases. The new device overcomes the weaknesses of intermittent monitoring, and might minimize damage to the respiratory tract mucosa even under conditions of sudden cuff pressure increases. These properties make it likely that the new device will be useful in clinical settings.

### Availability of supporting data

None

## Abbreviations

ANOVA: one-factor analysis of variance
CV: coefficient of variation
VAP: ventilator-associated pneumonia
VCV: volume-control ventilation
